# HIV, HCMV and mycobacterial antibody levels: a cross‐sectional study in a rural Ugandan cohort

**DOI:** 10.1111/tmi.13188

**Published:** 2018-12-27

**Authors:** Lisa Stockdale, Stephen Nash, Angela Nalwoga, Lorna Gibson, Hannah Painter, John Raynes, Gershim Asiki, Robert Newton, Helen Fletcher

**Affiliations:** ^1^ Faculty of Infectious and Tropical Diseases London School of Hygiene and Tropical Medicine London UK; ^2^ Faculty of Epidemiology and Population Health London School of Hygiene and Tropical Medicine London UK; ^3^ Medical Research Council/Uganda Virus Research Institute Entebbe Uganda; ^4^ African Population and Health Research Center Nairobi Kenya; ^5^ Department of Women's and Children's Health Karolinska Institutet Stockholm Sweden; ^6^ Department of Health Sciences University of York York UK; ^7^ International Agency for Research on Cancer Lyon France

**Keywords:** TB, HIV, HCMV, *Mycobacterium*, TB, VIH, HCMV, *Mycobacterium*

## Abstract

**Objectives:**

A growing evidence base implicates human cytomegalovirus (HCMV) as a risk factor for TB disease. We investigated total IgG and mycobacteria‐specific antibodies in a cross‐sectional study nested within a rural Ugandan General Population Cohort (GPC), in relation to HIV infection and the magnitude of HCMV IgG response.

**Methods:**

Sera from 2189 individuals (including 27 sputum‐positive TB cases) were analysed for antibodies against mycobacteria (Ag85A, PPD, LAM, ESAT6/CFP10) and HCMV, tetanus toxoid (TT) and total IgG.

**Results:**

Anti‐mycobacterial antibodies increased with age until approximately 20 years, when they plateaued. Higher HCMV exposure (measured by IgG) was associated with lower levels of some anti‐mycobacterial antibodies, but no increase in total IgG. HIV infection was associated with a decrease in all anti‐mycobacterial antibodies measured and with an increase in total IgG.

**Conclusions:**

The increase in anti‐mycobacterial antibodies with age suggests increasing exposure to non‐tuberculous mycobacteria (NTM), and to *M.tb* itself. HIV infection is associated with decreased levels of all mycobacterial antibodies studied here, and high levels of HCMV IgG are associated with decreased levels of some mycobacterial antibodies. These findings point towards the importance of humoral immune responses in HIV/TB co‐infection and highlight a possible role of HCMV as a risk factor for TB disease.

## Abstract (fr)

**Objectifs:**

Des évidences croissantes impliquent le cytomégalovirus humain (HCMV) comme facteur de risque de la tuberculose (TB). Nous avons étudié les anticorps IgG totaux spécifiques des mycobactéries dans le cadre d'une étude transversale imbriquée dans une cohorte de population générale (CPG) ougandaise en zone rurale, en relation avec l'infection par le VIH et l'ampleur de la réponse des IgG anti‐HCMV.

**Méthodes:**

Des sérums provenant de 2.189 personnes (y compris 27 cas de TB à expectoration positive) ont été testés pour les anticorps anti‐mycobactéries (Ag85A, PPD, LAM, ESAT6/CFP10), et anti‐HCMV, pour l'anatoxine tétanique et les IgG totales.

**Résultats:**

Les anticorps anti‐mycobactériens augmentaient avec l’âge jusqu’à environ 20 ans où ils atteignent un plateau. Une exposition plus élevée au HCMV (mesurée par les IgG) était associée à des taux plus bas de certains anticorps anti‐mycobactériens, mais pas d'augmentation des IgG totales. L'infection par le VIH était associée à une diminution de tous les anticorps anti‐mycobactériens mesurés et à une augmentation des IgG totales.

**Conclusions:**

L'augmentation des anticorps anti‐mycobactériens avec l’âge suggère une exposition croissante aux mycobactéries non tuberculeuses (MNT) et à *M. tuberculosis* lui‐même. L'infection par le VIH est associée à une diminution des taux de tous les anticorps anti‐mycobactériens étudiés ici, et des taux élevés des IgG anti‐HCMV sont associés à une diminution des taux de certains anticorps anti‐mycobactériens. Ces résultats soulignent l'importance des réponses immunitaires humorales dans la coinfection VIH/TB et mettent en évidence le rôle possible du HCMV comme facteur de risque de la maladie TB.

## Introduction

The role of antibody‐mediated immunity in TB has not been fully elucidated (reviewed in 1). Despite the importance of cell‐mediated Th1 responses in TB disease 2, 3, attempts to stimulate this arm of the human immune response by vaccine developers have not translated into protection from disease in human efficacy trials 4, 5, and there may be useful humoral correlates of disease or protection yet to be identified.

A growing evidence base for the importance of antibodies in protection against other intracellular pathogens such as *Listeria monocytogenes* 6 and *Salmonella* spp. 7 has led to renewed interest in humoral responses to TB and the factors which may affect them. Recent work has suggested a potential protective role of antigen 85A (Ag85A)‐specific antibodies in reduced TB disease risk in BCG‐vaccinated South African infants 8, 9.

It is known that TB and HIV work synergistically to exacerbate morbidity and mortality in co‐infected individuals with respect to both diseases 10. Epidemiologically, concomitant viral infections other than HIV are known to be associated with poor TB outcomes. In Taiwan Hepatitis C infection was associated with a higher risk of developing active TB disease 11, and in South Africa influenza/TB co‐infection was associated with increased mortality 12. A large TB vaccine trial in South Africa, which investigated correlates of TB disease risk or protection, found an association between CD8 T‐cell activation and HCMV response 8 that was linked to an increased risk of TB disease and shorter time to diagnosis 13.

The ubiquitous herpes virus HCMV is known to cause immune activation 14, immune senescence 15, and is a significant factor in immune variation 16. Recently, our group has reported elevated levels of HCMV IgG among TB patients compared to controls 17. Despite this, and the findings of some early epidemiologic studies 18, 19, data linking HCMV and TB are sparse.

To investigate mycobacteria‐specific antibody levels across ages, and to examine potential effects of HCMV co‐infection on antibody levels, this study tested 2187 stored serum samples (of which 27 were active TB cases) from a rural Ugandan cohort for IgG responses to Ag85A, purified protein derivative (PPD), lipoarabinomannan (LAM) and CFP10/ESAT6, along with IgM responses to Ag85A. These antigens were chosen based on availability, evidence of their potential importance in TB disease from the literature (Ag85A 9, LAM 20, 21, PPD 22), and specificity to *M.tb* (CFP10/ESAT6 23). Seropositivity to HCMV was measured and existing data on HIV, BCG vaccination status, as well as demographic information was matched and investigated for associations. Responses to tetanus toxoid (TT) and total IgG levels were also investigated as control antibodies.

## Materials and methods

### Study area and design

The General Population Cohort (GPC) is a population‐based open cohort study in rural south‐western Uganda, administered by the Medical Research Council (MRC) UK in collaboration with the Uganda Virus Research Institute (UVRI) 24. The cohort comprises a cluster of 25 neighbouring villages with approximately 20 000 residents (52% aged <13 years) from three ethnic groups, the majority (75%) being from the Baganda tribe, the main tribal group in the region. Data are collected through an annual census, questionnaire and serological survey (further details on questions included in the census can be found in a publication by Asiki *et al*. 24). Demographic information was collected at the same time as the blood samples. Blood specimens were obtained and immediately tested for HIV‐1. Samples with inconclusive results were retested. Remaining sera were stored at −80 °C. All samples used for this study, except active TB cases, were collected from adults and children in GPC round 22, conducted in 2011. Active TB cases were diagnosed through positive sputum smear microscopy after passive case detection and were sampled from a range of GPC rounds between 1999 and 2014. Sera collected as close as possible to the time of TB diagnosis were used for this study (between 5 years prior to, and 0.8 years after diagnosis of active TB diagnosis).

### Sampling

Individuals were selected for inclusion in this cross‐sectional study at random after stratification by age and sex. Due to anticipated high levels of HCMV seropositivity, infants under the age of 5 were oversampled. Individuals were only sampled once and siblings and parent‐child pairs were not excluded. The total planned sample size was 2000, plus 10% oversampling. A target of approximately 100 individuals per year of age up to 5 years, 200 individuals aged 6–10, 11–15 and 16–20, 200 in subsequent 10‐year intervals up to 60 years and 200 people aged 61 years or over. The sex ratio was approximately equal within each age group (Table 1).

**Table 1:** Characteristics of study participants (HCMV seropositive individuals by age group (*n* = 1988)). Sex, HIV infection, BCG vaccination and TB case prevalence is shown for each age group

Age group (year)	*n*	Sex (percentage female (number/total))	HIV prevalence, percentage (number/total)	BCG vaccinated, percentage (number/total)	Active TB cases, percentage (number/total)
<1	75	47% (35/75)	4% (3/73)	76% (54/71)	0% (0/75)
1	83	51% (42/83)	1% (1/80)	96% (73/76)	0% (0/83)
2	82	52% (43/82)	1% (1/78)	90% (63/70)	0% (0/82)
3	87	47% (41/87)	1% (1/85)	93% (70/75)	0% (0/87)
4	100	52% (52/100)	0% (0/97)	94% (82/87)	0% (0/100)
5	106	54% (57/106)	1% (1/101)	8% (80/90)	0% (0/106)
6–10	183	47% (86/183)	1% (2/175)	90% (135/150)	0% (0/183)
11–15	192	49% (94/192)	0% (0/187)	81% (125/155)	1% (1/192)
16–20	204	51% (105/204)	1% (3/204)	69% (132/190)	0.5% (1/204)
21–30	216	56% (120/216)	9% (19/216)	70% (128/184)	2% (5/216)
31–40	238	52% (123/238)	13% (31/238)	57% (119/209)	4% (10/238)
41–50	166	45% (74/166)	15% (25/166)	52% (75/145)	4% (6/166)
51–60	109	49% (53/109)	6% (7/109)	53% (52/98)	4% (4/109)
61+	147	46% (68/147)	1% (2/147)	26% (37/144)	0% (0/147)
Total	1988	50% (993/1988)	5% (96/1956)	70% (1225/1744)	1.4% (27/1988)

### Ethics

Written consent or assent in conjunction with parental/guardian consent for those younger than 18 years were obtained following Uganda National Council of Science and Technology (UNCST) guidelines before all survey procedures 24. Written consent/assent was also obtained from participants on the use of their clinical records and stored biological samples for research purposes.

Ethical approval for the use of GPC samples for this study was obtained from the UVRI Research and Ethics Committee and from the UNCST, in addition to the London School of Hygiene & Tropical Medicine (LSHTM), London, UK.

### Serology: Ag85A, PPD, LAM, CFP10/ESAT6 and TT

Antibody content was measured using ELISA. Experimenters were blind to exposure group for all samples. Testing was conducted at UVRI in Entebbe, Uganda (Ag85A IgG and PPD IgG) and LSHTM, London, UK (CFP10/ESAT6 IgG, Ag85A IgM, LAM IgG and TT IgG). Half volume ELISA plates (Fisher Scientific) were coated overnight at 4 °C with sodium carbonate buffer containing 1.5 μg/ml recombinant Ag85A protein (Aeras, USA), 1.5 μg/ml PPD (Lot 051815KA, Aeras, USA), 0.5 μg/ml TT (02/232, NIBSC, UK), 0.5 μg/ml LAM (NR‐14848, BEI Resources, VA, USA), 0.25 μg/ml each CFP10 (NR‐49425, BEI Resources, VA, USA) and ESAT6 (NR‐14868, BEI Resources, VA, USA). Plates and reagents were brought to room temperature and the plate was washed three times with PBS, 5% Tween20 (v/v) (PBST). After blocking with PBS 5% milk (w/v) blocking buffer, duplicate 1:100 test serum, high and low PPD responder controls and plate blanks consisting of assay diluent alone with no serum were added in triplicate. Incubation for 2 h at room temperature and washing with PBST was followed by a one hour incubation with an appropriately diluted peroxidase‐conjugated secondary antibody in PBST‐5% (IgG, 1:500 dilution of goat anti‐human IgG‐HRP (04‐10‐20, KPL, USA); IgM, 1:10 000 dilution of goat anti‐human IgM‐HRP (Abcam ab97205, RRID: AB_10695942)). After washing with PBST, 50 μl tetramethylbenzidine (TMB, BD BioSciences, USA) was added to each well. The plate was incubated for 15 min in the dark before the reaction was stopped by adding 50 μl of 2M Sulphuric Acid (Sigma, USA) to each well. Absorbance was measured at 450 nm within 30 min to obtain optical density (OD), a surrogate marker of antibody titre.

**Figure 1:**
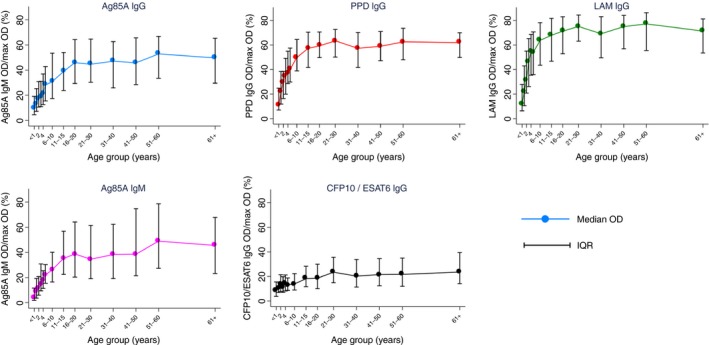
Median mycobacterial antibody OD by age group. Vertical lines show IQR for median values for each age group. Total *n* = 1988. OD values rescaled to the percentage of maximum for graphical representation. IQR, interquartile range, OD, optical density. [Colour figure can be viewed at wileyonlinelibrary.com]

**Figure 2:**
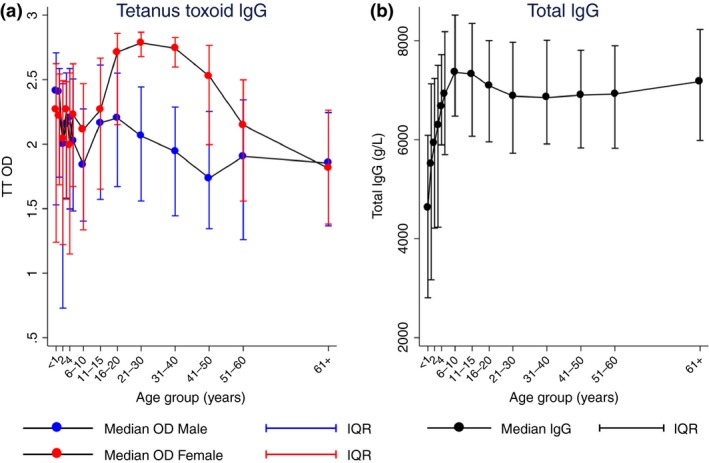
(a) Median OD levels for TT IgG by sex and age group. (b) Median total IgG (g/l) by age group. Vertical lines show IQR for each median data point. Total *n* = 1988. Figure 2a red lines represent median values for females, blue lines for males. Figure 2(b) shows data for males and females combined. IQR, interquartile range, OD, optical density. [Colour figure can be viewed at wileyonlinelibrary.com]

Mean blank values were subtracted from all OD readings, and geometric means of duplicates were used to reduce the effect of skewed distribution. Median levels with interquartile ranges (IQR) are used for graphical representation of antibody levels in Figures 1 and 2.

### Total IgG serology

All samples were tested for total IgG content at LSHTM. As above, half volume ELISA plates were coated overnight with mouse anti‐human IgG at 0.5 μg/ml (Abcam ab200699). IgG antibody standards (134.4–8.4 ng/ml) were prepared by diluting purified human IgG (Sigma I4506, RRID:AB_1163606) in PSBT‐5% milk and test sera were diluted 8 × 10^5^ in PBST‐5% Milk. Duplicate test samples, controls and blanks were incubated for one hour at 37 °C, and, after washing, samples incubated with peroxidase‐conjugated goat anti‐human Fc (Abcam ab97225, RRID: AB_10680850) diluted 1/500 with PBST‐5% Milk for one hour at room temperature. Plates were developed with TMB, and sulphuric acid was added to stop the reaction. Plates were read at 450 nm and OD measurements converted into g/l by use of the standard curve on each plate.

### Serology HCMV

Samples were tested for IgG antibodies against HCMV using a commercial ELISA kit (Novatec Immunodiagnostica GmbH) according to kit instructions (described in 17).

### Statistical analysis

Correlations between each pair of mycobacterial antibody OD levels were conducted using Spearman's rho. Because of the ubiquity of HCMV infection within this population 17, analysis was conducted on the HCMV‐seropositive population only.

Individuals seropositive for HCMV IgG were further categorised into three groups according to the tertiles of HCMV antibody concentrations (measured by OD): low, medium and high.

Linear regression was used to determine the association of each antibody response (as continuous dependent variables) with HCMV IgG tertile and HIV infection (as independent variables with HCMV as a categorical variable), adjusting for age and sex. BCG vaccination status and active TB disease were also included in the regression model, however as 89% (24/27) of active TB cases had unknown BCG status, it was not possible to include both variables in the same model. The model including TB was preferred, with the model including BCG run as a comparison to confirm the consistency of the results. BCG vaccination was included in a separate regression analysis using the same model.

Individuals with unknown HIV status were excluded from all regression analyses and individuals with unknown BCG vaccination status were excluded from regression analyses including BCG. To account for evidence of non‐linearity of antibody OD with age (likelihood ratio test *P* < 0.001 for all antibodies), a quadratic term for age was included in the regression analyses. Numbers included in each of the regression analyses are shown in Tables 2 and 3.

**Table 2:** Multivariable linear regression of variables with a potential influence on mycobacterial antibody levels. Values obtained using a multivariable regression model including age, quadratic age, sex, HIV and TB status, with *P* values and 99% CI (*n* = 1956)

Variable	*n* (%)	Ag85A IgG OD		PPD IgG OD		LAM IgG OD		CFP10 ESAT 6 IgG OD		Ag85A IgM OD	
		Coeff (99% CI)	*P* value	Coeff (99% CI)	*P* value	Coeff (99% CI)	*P* value	Coeff (99% CI)	*P* value	Coeff (99% CI)	*P* value
Sex											
Male	978/1956 (50.0%)	Baseline		Baseline		Baseline		Baseline		Baseline	
Female	978/1956 (50.0%)	−0.002 (−0.045, 0.041)	0.9	−0.002 (−0.042, 0.038)	0.91	−0.017 (−0.099, 0.064)	0.58	−0.025 (−0.083, 0.033)	0.27	−0.012 (−0.103, 0.079)	0.73
HCMV tertiles											
HCMV low	650/1956 (33.2%)	−0.037 (−0.090, 0.016)	0.07	−0.009 (−0.051, 0.033)	0.57	−0.061 (−0.151, 0.030)	0.08	−0.100 (−0.156, −0.044)	<0.001	−0.104 (−0.205, −0.004)	0.01
HCMV medium	650/1956 (33.2%)	Baseline		Baseline		Baseline		Baseline		Baseline	
HCMV high	656/1956 (33.5%)	−0.067 (−0.122, −0.012)	<0.001	−0.057 (−0.105, −0.009)	<0.001	−0.107 (−0.194, −0.019)	<0.001	−0.015 (−0.077, 0.047)	0.54	−0.058 (−0.152, 0.035)	0.11
HIV status											
Negative	1860/1956 (95.1%)	Baseline		Baseline		Baseline		Baseline		Baseline	
Positive	96/1956 (4.9%)	−0.240 (−0.361, −0.119)	<0.001	−0.310 (−0.422, −0.198)	<0.001	−0.420 (−0.586, −0.253)	<0.001	−0.266 (−0.409, −0.123)	<0.001	−0.446 (−0.640, −0.251)	<0.001
TB status											
Negative	1929/1956 (98.6%)	Baseline		Baseline		Baseline		Baseline		Baseline	
Positive	27/1956 (1.4%)	−0.101 (−0.322, 0.121)	0.24	−0.035 (−0.217, 0.147)	0.62	0.158 (−0.107, 0.424)	0.13	0.175 (−0.241, 0.590)	0.28	−0.135 (−0.609, 0.340)	0.46

**Table 3:** Multivariable linear regression of variables with a potential influence on total IgG and TT‐specific IgG levels. Values obtained using a multivariable regression model including age, quadratic age, sex, HIV and TB status, with *P* value and 99% CI (*n* = 1956)

Variable	*n* (%)	Total IgG (g/l)		TT IgG OD	
		Coeff (99% CI)	*P* value	Coeff (99% CI)	*P* value
Sex					
Male	978/1956 (50.0%)	Baseline		Baseline	
Female	978/1956 (50.0%)	−0.326 (−2.350, 1.697)	0.68	0.272 (0.188, 0.356)	<0.001
HCMV tertiles					
HCMV low	650/1956 (33.2%)	0.318 (−2.240, 2.877)	0.75	−0.090 (−0.187, 0.008)	0.02
HCMV medium	650/1956 (33.2%)	Baseline		Baseline	
HCMV high	656/1956 (33.5%)	0.915 (−1.508, 3.338)	0.33	0.065 (−0.029, 0.159)	0.08
HIV status					
Negative	1860/1956 (95.1%)	Baseline		Baseline	
Positive	96/1956 (4.9%)	9.110 (3.735, 14.485)	<0.001	−0.172 (−0.357, 0.012)	0.02
TB status					
Negative	1929/1956 (98.6%)	Baseline		Baseline	
Positive	27/1956 (1.4%)	−5.789 (−13.298, 1.720)	0.05	−0.081 (−0.328, 0.165)	0.4

CI, confidence interval; OD, optical density.

To account for multiple comparisons, 99% confidence intervals (CIs) are reported and a *P* value of 0.01 was considered to represent strong evidence to reject the null hypothesis. Due to different dynamic ranges of spectrophotometers used in Uganda and the UK, antibody OD measurements (for mycobacterial antibodies only) were rescaled for graphical representation. This was done by subtracting the minimum value within the range from each value, dividing by the range for that antibody and multiplying by 100; hence Figure 1 shows the percentage of the maximum OD. All analyses were performed on raw data using Stata version 14 (Stata Corporation, College Station, TX, USA). Anonymised participant data are available.

## Results

Sera were sampled from 2189 individuals. Total IgG levels could not be determined in 15 instances, likely due to protein degradation. Of the remaining 2174 individuals, 8.6% (186/2174) were HCMV‐seronegative and were excluded from analysis. The HCMV‐negative population contained no TB cases and 4 HIV‐positive individuals. Ninety‐eight (53%) were female and the mean age was 14.6 years (range 3 months–95 years).

The remaining 1988 individuals were included in further analyses. The mean age was 23.4 years (range 30 days–100 years) and 50% were female (Table 1). Ninety‐eight per cent (1956/1988) of individuals had a conclusive HIV result, and of those, 4.9% (96/1956) were HIV‐positive, with the greatest proportion in the 31–50 year age group.

Twenty‐seven sputum‐confirmed TB cases were included in the study, of whom 63% (17/27) were female and whose mean age was 36 years (range 12–59 years). Only three TB cases had BCG vaccination information and all of those were unvaccinated. All 27 were HCMV‐seropositive, with 59% (16/27) having HCMV IgG levels in the upper tertile.

### Antibody responses

IgG against Ag85A, PPD, LAM, CFP10/ESAT6 and IgM against Ag85A all increased from birth to approximately 20 years of age, after which OD values reached a plateau. Figure 1 shows median OD for each of the mycobacterial antibody levels by age group. Initial levels in infants under the age of one year range from a median of 4% of maximum OD (0.12 OD) for Ag85A IgM to 12% of maximum OD (0.35 OD) for LAM IgG. Responses to the *M.tb*‐specific ESAT6/CFP10 antigens did not show the same rapid increase from birth as responses to other mycobacterial antibodies. The highest 10% of responders in terms of CFP10/ESAT6, being *M.tb* specific antigens, were investigated further and there was no evidence of a difference in proportion of TB cases or HIV positive individuals compared with the lowest 10%.

Univariate analysis showed no differences in mean OD levels by sex. Analysis of correlation showed a high degree of co‐linearity between all mycobacterial antibodies (0.33> rho <0.65, all *P* < 0.001). OD ranged from 0.002 to 1.793 OD for Ag85A IgG, 0.000 to 1.930 OD for PPD IgG, 0.004 to 2.961 OD for LAM IgG, 0.005 to 2.927 OD for CFP10/ESAT6 IgG and 0.001 to 2.926 OD for Ag85A IgM.

Tetanus toxoid and total IgG levels were investigated as control antibodies (Figure 2). TT vaccination induces high levels of TT IgG and is given at birth and to pregnant females as part of the Uganda immunisation schedule 25. TT IgG showed high levels of antibody from birth and a clear sex difference with females having higher overall IgG levels (*P* < 0.001, Figure 2a). This difference is driven by females aged between 16 and 50 years (Figure 2a). Total IgG levels increase with age from birth (median 46.2 g/l in infants less than one year of age) to a plateau after 11 years of age (median 69.4 g/l), Figure 2b. Since a univariate analysis of total IgG by sex indicated no difference, data were not separated by sex for this plot.

**Figure 3:**
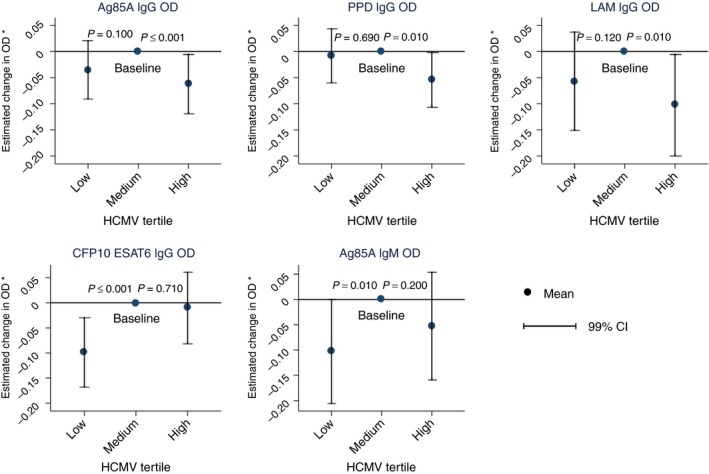
Adjusted mean OD change associated with HCMV tertile comparing to medium HCMV tertile as baseline. *P* values from multivariable linear regression model including age, quadratic age, sex, HIV and TB. Total *n* = 1956. Vertical lines show 99% CI for each mean OD change. CI, confidence interval, OD, optical density (*model includes, age, quadratic age, sex, HIV and TB). [Colour figure can be viewed at wileyonlinelibrary.com]

In a multivariable linear regression model including TB disease status, being HIV‐positive was associated with a decrease in all mycobacterial antibody levels studied (magnitude ranged from mean decrease in 0.240 OD (99% CI −0.361, −0.119) for IgG against Ag85A), to a mean decrease in 0.446 OD (99% CI −0.640, −0.251) for IgM against Ag85A, all *P* < 0.001, Table 2). Similarly, being in the upper tertile of HCMV IgG response was associated with a decrease in Ag85A, PPD and LAM IgG levels compared to being in the middle tertile: a mean decrease in 0.067 OD (99% CI −0.122, 0.012) for Ag85A IgG, 0.057 OD (99% CI −0.105, −0.009) for PPD IgG and 0.107 (99% CI −0.194, 0.019) for LAM IgG (all *P* < 0.001, Table 2). While the direction of change was the same for CFP10/ESAT6 IgG and Ag85A IgM, the mean decrease associated with being in the upper tertile of HCMV IgG response was not sufficiently large to provide good evidence of a difference based upon our significance threshold (Table 2). The association between mycobacterial antibody levels and magnitude of HCMV IgG response was not linear (Figure 3): in comparison to the middle tertile of HCMV IgG response, the lowest tertile was also associated with a decrease in mycobacterial antibody levels. A mean decrease of 0.100 OD (99% CI −0.156, −0.044) *P* < 0.001 was seen for CFP10/ESAT6 IgG and a mean decrease of 0.104 OD (99% CI −0.205, −0.004) *P* = 0.01 for Ag85A IgM. The direction of change was the same for the remaining mycobacterial antibodies (Table 2).

Being a sputum‐confirmed active TB case was not associated with any differences in any mycobacterial antibody level after adjusting for age, sex, HIV and HCMV level, although given that there were only 27 cases, there was limited statistical power to detect associations (Table 2). Coefficients of the effect of HIV and HCMV for each outcome measurement were similar for a model including BCG instead of TB, and the magnitude of effect was not altered by adjusting for total IgG. Being BCG vaccinated was associated with a 0.115 OD increase in TT IgG (99% CI 0.004–0.227) *P* = 0.01.

Table 3 shows that HIV positivity was associated with an increase in total levels of IgG (9.110 g/l (99% CI 3.735, 14.485), *P* < 0.001). Being female was associated with an increase in TT of 0.272 OD (99% CI 0.188, 0.356, *P* < 0.001) but no difference in total IgG levels. While being an active TB case was not associated with any differences in TT IgG, being a TB case was associated with a 5.8 g/l decrease (99% CI −13.298, 1.720) in total IgG however this was not significant (*P* = 0.05). The magnitude of HCMV IgG response did not affect either total IgG levels or TT IgG response (Table 3).

## Discussion

In this study, we found that HIV infected individuals had decreased levels of all mycobacterial antibodies studied, with a concomitant increase in total IgG. We also found that individuals with the highest levels of HCMV IgG (indicative of highest exposure 26, 27), had decreased levels of IgG specific for Ag85A, PPD and LAM, but had no corresponding change in total IgG levels. Previously our group has shown that TB patients have increased levels of HCMV IgG 17 and that HCMV positive infants (as measured by HCMV IFN‐*γ* ELISPOT) were at higher risk of progressing to active TB disease 13. HCMV infection, being linked to poor long‐term health outcomes 28, 29 and large immune system subversion 16, 30, may be acting to manipulate mycobacterial antibodies which may be contributing to protective mechanisms against *M.tb* infection.

As has been seen previously 31, humoral responses against RD1 antigens ESAT6 or CFP10 (mycobacterial proteins associated specifically with *M.tb* and not NTM or BCG) did not show an association with active TB cases in this study. Due to a lack of active TB case‐finding in the GPC, it is likely that some of the individuals classed as TB negative here were latently infected with *M.tb* based on epidemiologic data on exposure in Uganda 32, 33. The finding that IgG responses to these *M.tb*‐specific antigens increase with age, following a similar pattern as other non‐*M.tb*‐specific mycobacterial antibodies, suggests that while NTM may be driving high responses of acute (IgM) and sustained (IgG) mycobacterial responses, exposure to *M.tb* itself cannot be ignored.

BCG vaccine status allocation in this study was by the presence of scar, inspection of immunisation card or verbal confirmation by parent of guardian. Despite issues with these methods 34, classification was thought to be robust due to the evidence of association of TT IgG with BCG indicating that people vaccinated for one Extended Programme of Immunization (EPI) schedule vaccine are more likely to have received other vaccinations upon accessing local health centres.

The magnitude of difference in mycobacterial antibodies seen due to HIV infection was far greater than due to either active TB disease or BCG vaccination. The lower levels of mycobacterial antibodies seen in individuals infected with HIV is accompanied by an increase in total IgG. The effect seen here points towards HIV infection having a mycobacteria‐specific effect as opposed to a more general depression of all antibody levels, thereby confirming evidence seen previously in a much smaller number of patients in Italy 35. It is known that HIV preferentially depletes TB‐specific CD4 T cells 36. Through their absence, TB‐specific T cells would not provide signals to B cells in order for them to proliferate and differentiate into immunoglobulin‐secreting plasma cells. As far as we are aware, the possible downstream humoral mechanism of HIV‐associated TB‐specific T‐cell depletion has not been investigated.

Here we see a similar association between the highest levels of HCMV IgG, and decreased levels of some mycobacterial antibodies. This is not accompanied by a decrease in total IgG or TT IgG. As a human herpes virus, HCMV persists in a variety of cell types in a dormant state and is transmitted through body fluids 37. Infection is not normally associated with symptomatic disease; however, it poses a huge burden on the immune system, with maintenance of up to 30% of both CD4 and CD8 circulating memory T cells being specific to HCMV 38. The virus is a significant factor in immune variation 16, immunosenescence 15 and causes immune activation 39, including non‐specific polyclonal B‐cell activation and proliferation 40.

In this study, we see decreased mycobacterial antibody levels at both HCMV IgG extremes among HCMV seropositive individuals. Evidence of HCMV effect on vaccine responses exists, showing both a detrimental impact of HCMV infection 41, 42 and a positive, ‘adjuvanting’, effect of HCMV on vaccine‐induced antibody responses in humans 43. Given reports that cellular responses to heterologous virus challenge in mice may depend on the initial viral dose of HCMV 44, 45 the lack of a linear response between HCMV IgG and mycobacterial antibody levels seen in this study may also be indicative of different effects at different HCMV exposure levels. PBMC samples were not available from this cohort for us to determine if the magnitude of HCMV IgG level had a similar impact on the mycobacteria‐specific cellular immune response. However, it is likely that cellular immunity is also impacted by HCMV given that HCMV has been shown to affect both humoral and cell‐mediated immune responses to vaccine antigens 46, 47.

Before the widespread use of highly active anti‐retrovirals (HAART), HCMV/HIV co‐infection saw HCMV as an important cause of severe non‐AIDS events, including death, in HIV‐infected individuals 48. High levels of HCMV IgG, caused by repeated reactivation or reinfection events, are associated with higher all‐cause mortality 49 and lower CD4 cell count, with accompanying worse response to anti‐retrovirals 50. Despite literature describing HCMV‐induced B‐cell activation 40, 51, 52, in this study we saw no relationship between HCMV exposure and increased levels of total IgG. We do however see a trend towards decreased mycobacterial antibody response in individuals with very high HCMV IgG responses after adjusting for HIV infection. Considering the risk of developing active TB disease increases over 20 times in individuals with HIV 53, it is likely that any added effect of HCMV would be masked. The mycobacterial antibody‐specific decrease seen with both HIV infection and, independently, in the highest tertile of HCMV exposure, may be indicative of a functional, protective role of mycobacterial‐specific antibodies which are independently decreased by HIV and high levels of exposure to HCMV.

In summary, both HIV infection and high levels of HCMV IgG were both associated with decreases in mycobacterial antibody levels. Given this novel finding of effect of HCMV exposure on mycobacterial antibody levels, investigation of magnitude of HCMV may be important in future TB clinical trials to understand the immune environment, independently of HIV infection status. In addition to quantification of antibody levels, we believe that it will be important to measure HCMV effect on mycobacteria‐specific cellular responses. A more nuanced understanding of the quality, as well as the quantity, of the mycobacterial antibodies elicited, in terms of antibody class, subtype and avidity may help to shed light upon a potential mechanism by which they might confer protection to host cells.

Due to insufficient volumes of sera, we were unable to investigate HCMV viral load in serum samples to ascertain whether active infection at point of blood draw was associated with any of the measurements taken for this study. Despite this, we believe that the use of HCMV‐specific IgG is a robust a measure of cumulative exposure. As previously mentioned, active TB case finding is not carried out within the GPC and therefore we cannot be sure that some individuals analysed as ‘non‐TB’ are not latently infected. PBMC samples were not available from this cohort for us to determine if HCMV had a similar impact on the mycobacterial cellular immune response.
